# Atypical Hemolytic Uremic Syndrome: Cancer-Induced or Chemotherapy-Induced?

**DOI:** 10.7759/cureus.13260

**Published:** 2021-02-10

**Authors:** Ashish Sethi, Raj Moses

**Affiliations:** 1 Internal Medicine, Allegheny Health Network, Pittsburgh, USA; 2 Oncology, Allegheny Health Network, Pittsburgh, USA

**Keywords:** atypical hemolytic uremic syndrome, eculizumab, hemolytic uremic syndrome, pancreas cancer, thrombotic microangiopathy, complement, gemcitabine-induced hemolytic uremic syndrome, gemcitabine, paroxysmal nocturnal hemoglobinuria (pnh), ravulizumab

## Abstract

Atypical hemolytic uremic syndrome (aHUS) is an atypical type of thrombotic microangiopathy (TMA), which is characterized by microangiopathic hemolytic anemia (MAHA), thrombocytopenia, and thrombi in small blood vessels, leading to end-organ damage. aHUS causes an over-activation of the complement pathway. There are many etiologies of aHUS, including inherited and acquired. This condition has a high mortality rate, as it is often detected late in the disease course. Eculizumab, an inhibitor of the terminal complement pathway, needs to be prescribed as soon as the diagnosis is confirmed. There is limited evidence, however, regarding the duration of treatment. Therefore, it is vital to conduct further analysis on other alternatives and pharmacokinetics with this type of complement inhibitor.

## Introduction

Hemolytic uremic syndrome (HUS), the most common subtype of thrombotic microangiopathy (TMA) is characterized by microangiopathic hemolytic anemia (MAHA), thrombocytopenia, and acute kidney injury. TMA are classified into three major categories, including Shiga toxin-producing Escherichia coli-hemolytic uremic syndrome (STEC-HUS), thrombotic thrombocytopenic purpura (TTP), and aHUS [1].

The most common preceding factor leading to typical HUS is infective diarrhea caused specifically by Shiga toxin-producing Escherichia (E.) coli (STEC ) or E. coli O157:H7. Conversely, aHUS has many possible etiologies, including malignancy, pregnancy, organ transplantation, human immunodeficiency virus (HIV), upper respiratory tract infections, non-E. coli diarrheal illnesses, and the use of certain drugs such as oral contraceptives (OCPs), ticlopidine, quinine, cyclosporine, tacrolimus. aHUS accounts for 5%-10% of all documented cases of HUS and is associated with a poor prognosis [2].

Our objective is to present a patient diagnosed with aHUS due to an underlying malignancy. Early diagnosis and treatment is vital, as aHUS can be life-threatening.

## Case presentation

**Figure 1:**
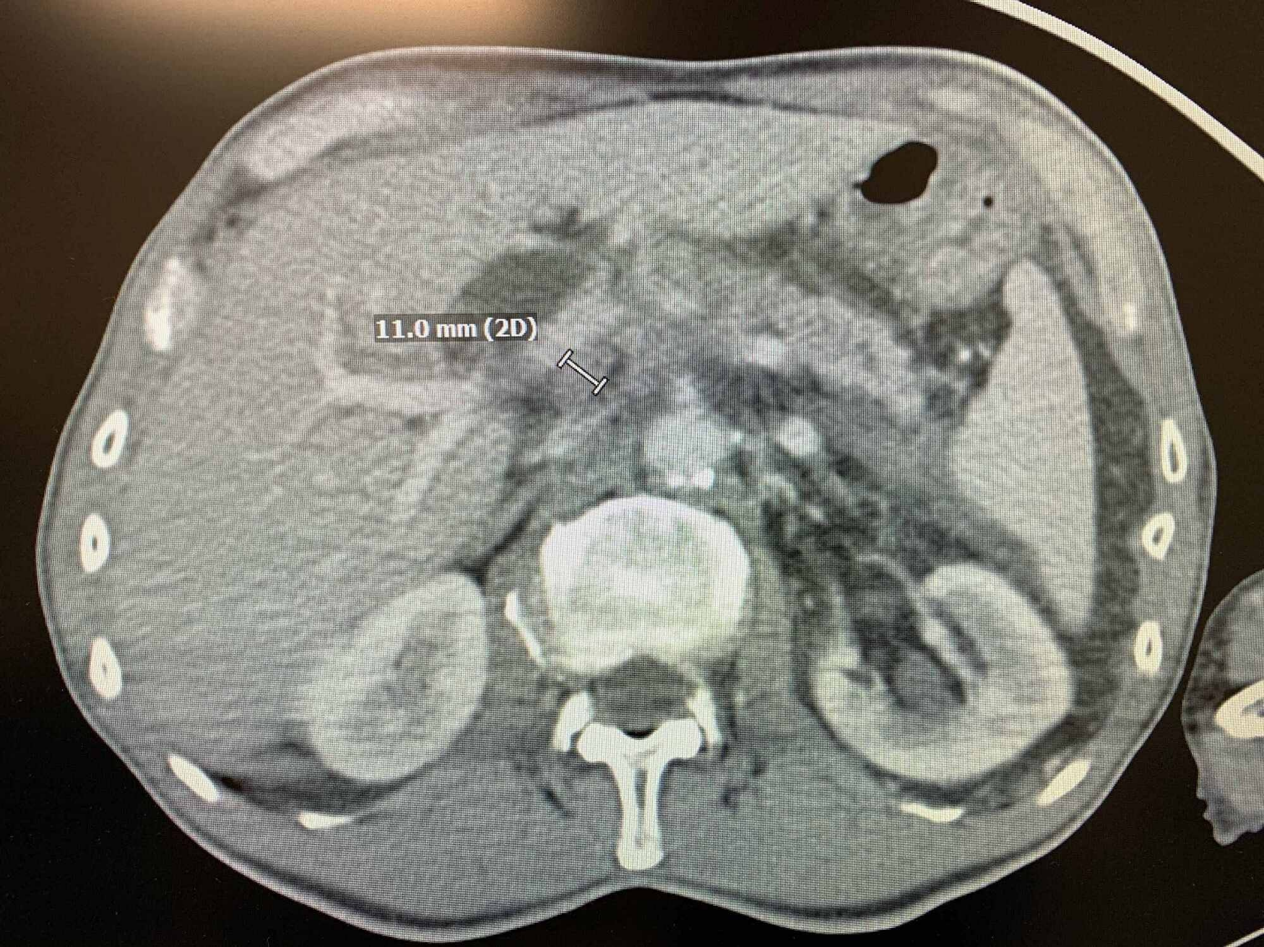
CT scan abdomen: CA pancreas Poorly delineated pancreatic head-neck junction mass measuring approximately 1.5 x 1 cm. There is ductal dilatation proximal to the mass up to 5 mm. Soft tissue density infiltration extending around the proximal portal vein, central superior mesenteric vein, central splenic vein, and proximal superior mesenteric artery is suspicious for locally advanced, unresectable disease. It also showed intra and extrahepatic biliary duct dilatation related to obstruction by the mass. CT: computed tomography; CA: Cancer

 A 67-year-old Caucasian male was diagnosed with an unresectable form of ductal adenocarcinoma of the pancreatic head (Figure 1) based on clinical impression and imaging modalities, including computed tomography (CT) scan and endoscopic retrograde cholangiopancreatography (ERCP). Prior to the diagnosis, he had persistent symptoms of infrequent vague abdomen pain for one year associated with early satiety, belching, and a seven to eight-pound weight loss.

Intravenous gemcitabine and albumin-bound paclitaxel chemotherapy regimens were administered for his stage T2a, N, M pancreatic cancer. After five months of gemcitabine therapy (cumulative dose 11,050 mg), the patient complained of severe fatigue, which was initially attributed to moderate anemia caused by the chemotherapy.

**Figure 2:**
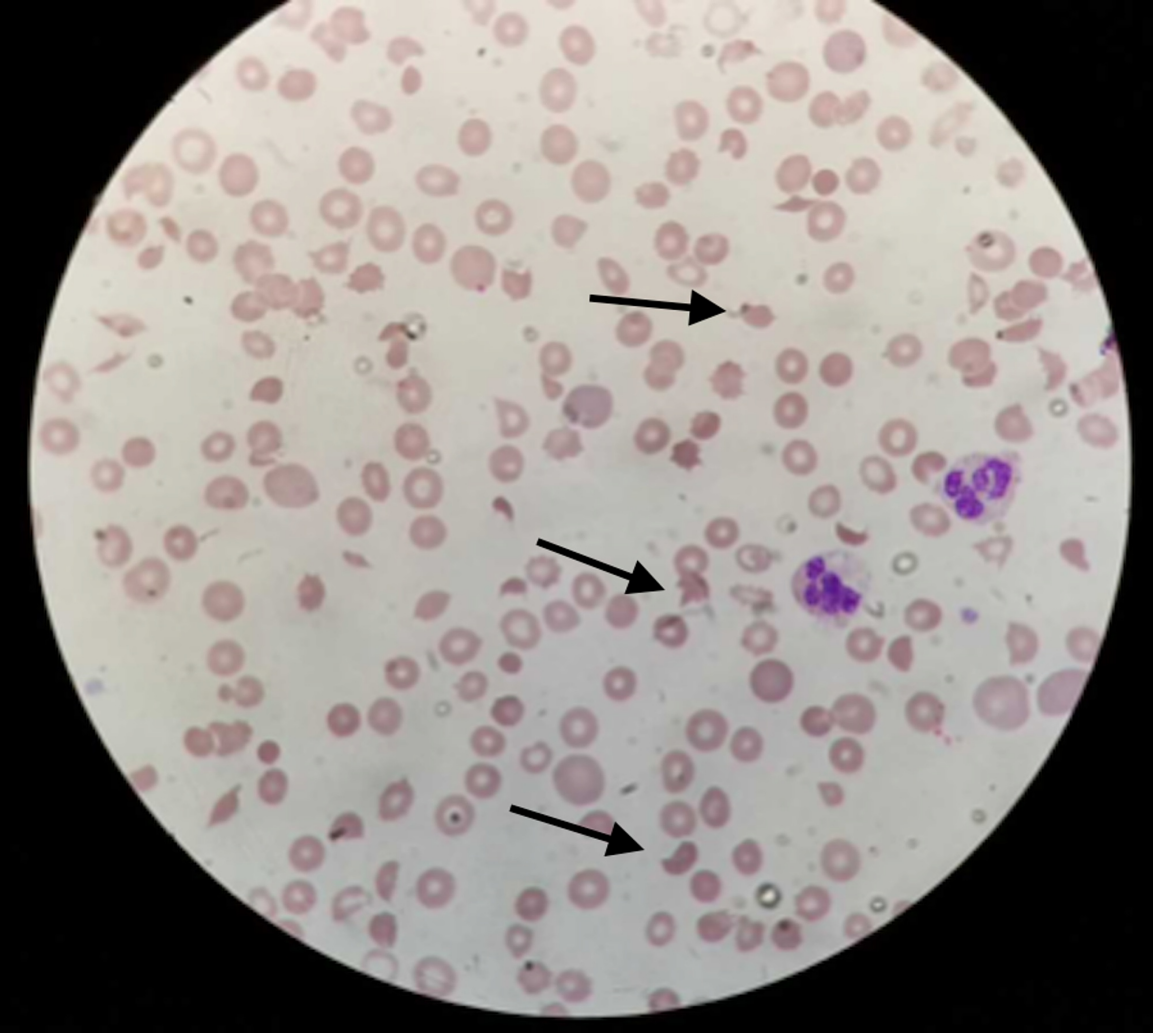
Peripheral blood film showing numerous schistocytes Permission was taken from the original publisher; adapted from Jain et al. [3]

**Figure 3:**
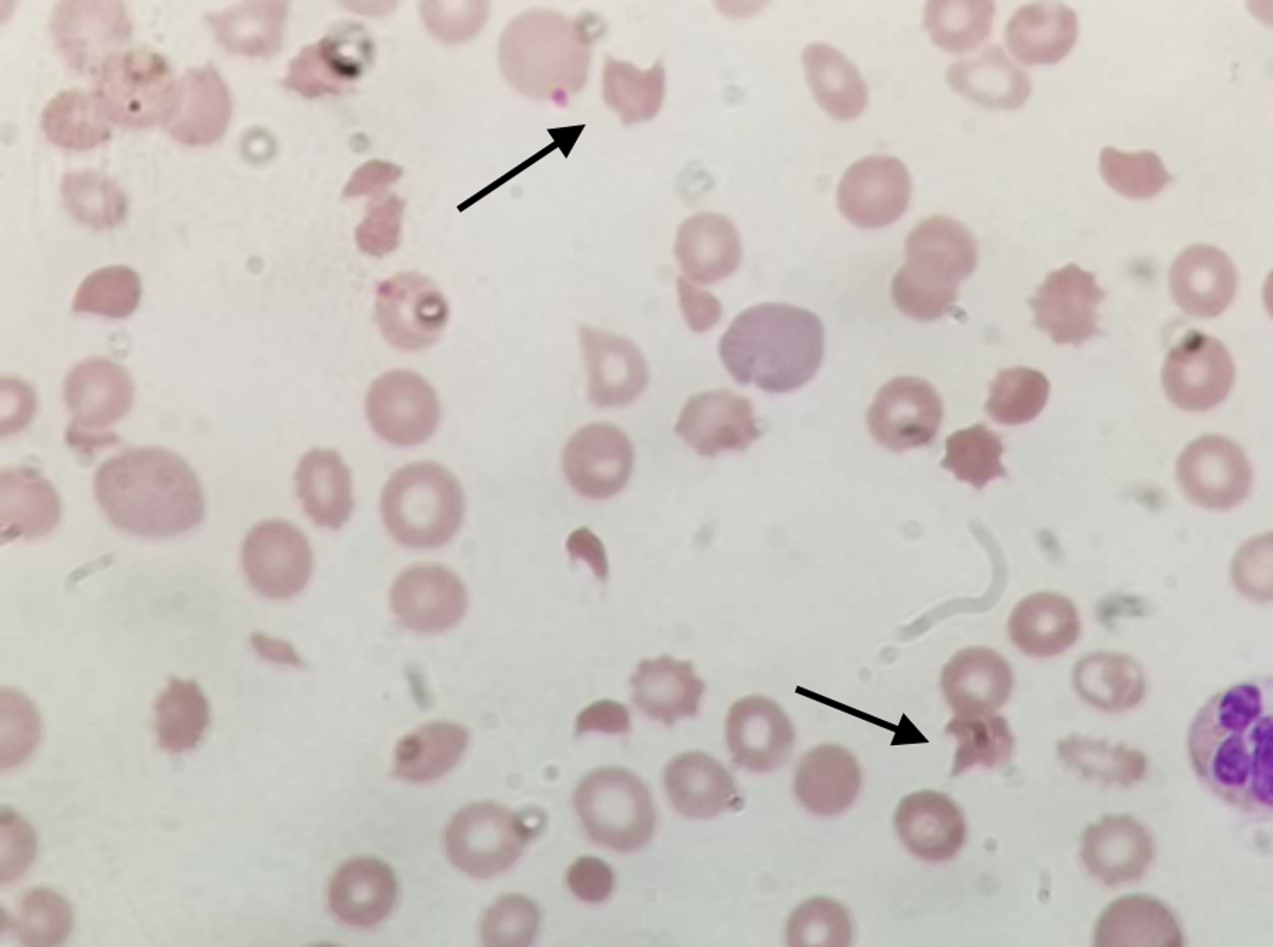
Schistocytes under magnification Permission was taken from the original publisher; adapted from Jain et al. [3]

A baseline complete blood count (CBC) and serum chemistry panel were within the normal range (serum creatinine 0.72 mg/ dL ). His serum creatinine increased steadily to 3.96 mg/dL after six cycles of single-agent gemcitabine (cumulative dose of gemcitabine 11,050 mg), and his platelet count dropped to 90k/mcl. Peripheral blood smear (PBS) showed rare schistocytes (Figure 2 and Figure 3). LDH was 514 IU/L and haptoglobin <10 mg/dL. His C3 and C4 levels were within normal limits. Urinalysis showed hematuria and 2+ protein. ADAMTS-13 (a disintegrin and metalloproteinase with a thrombospondin type 1 motif, member 13) activity was 53%. Direct and indirect Coomb’s test results were negative.

**Figure 4:**
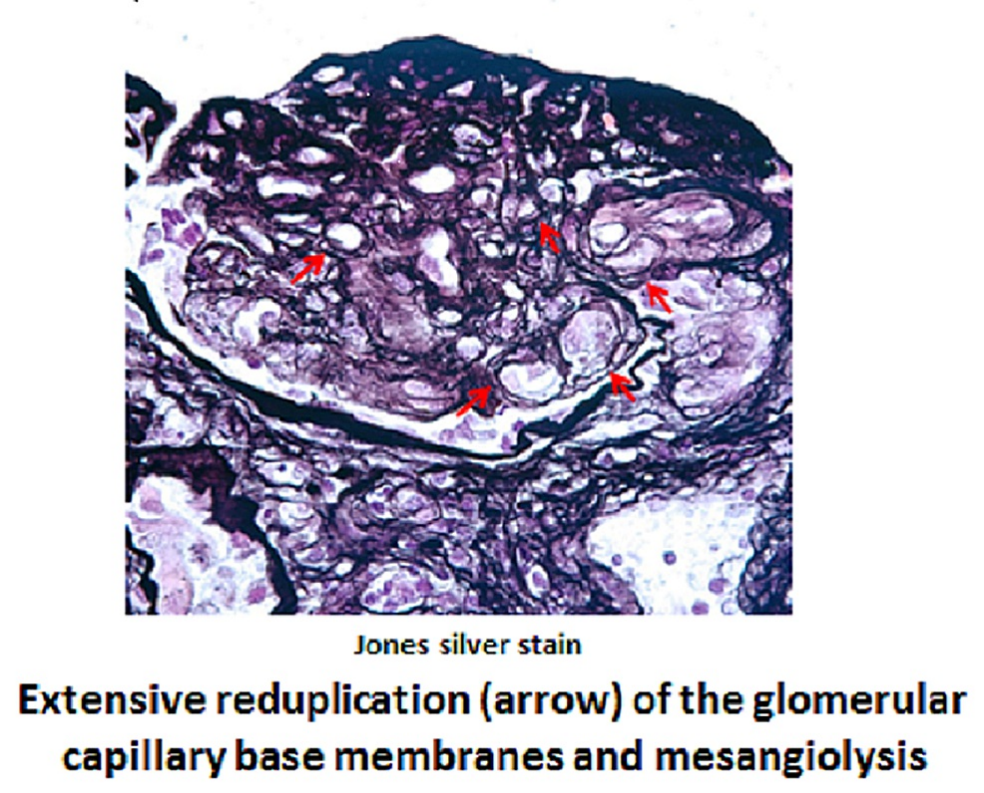
Kidney biopsy Jones silver stain

**Figure 5:**
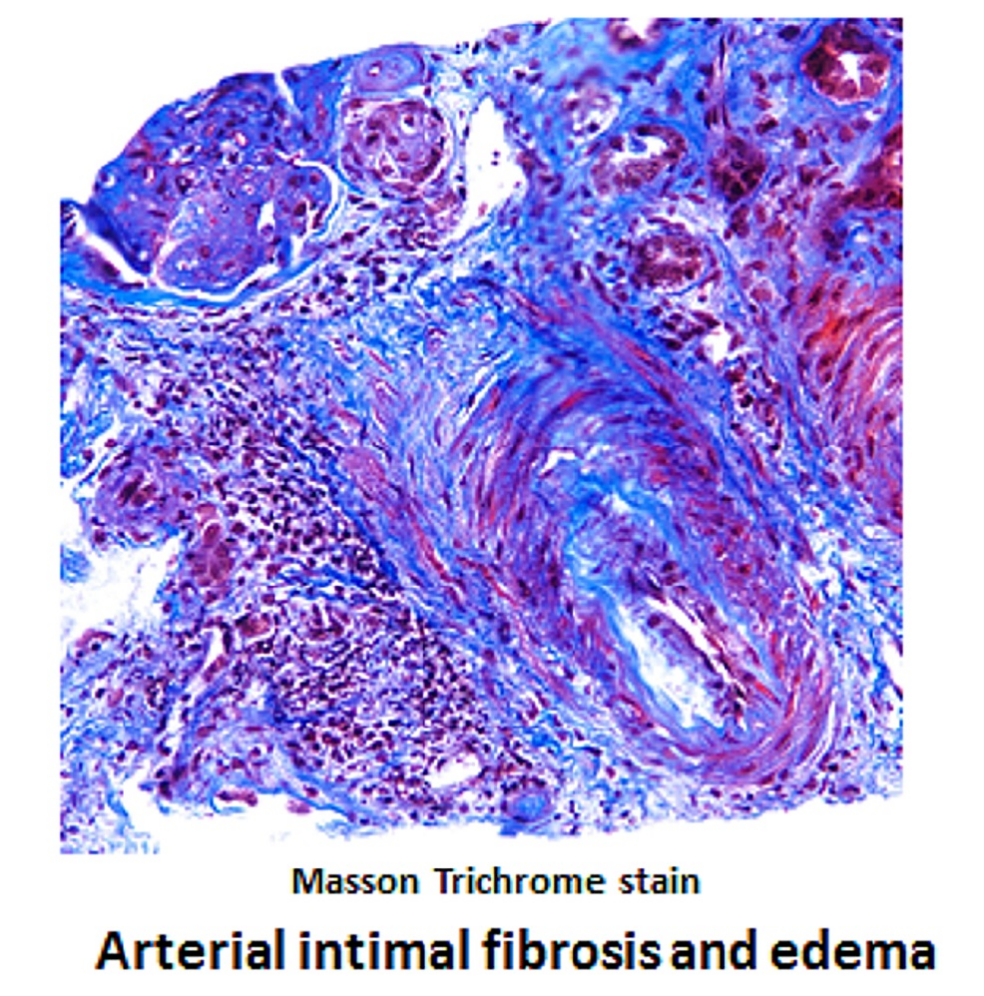
Kidney biopsy Masson trichrome stain

Gemcitabine was discontinued in view of possible TMA. There was no improvement in his hematologic or renal parameters for a few weeks after stopping the chemotherapy. A kidney biopsy was performed, which showed conclusive features of aHUS (Figures 4-5). ADAMTS13 activity assay values of 53% in our case ruled out TTP. Also, STEC-HUS as another differential diagnosis was excluded with no history of diarrhea and absence of Shiga toxin detection in the stool specimen.

Eculizumab 900 mg was administered subcutaneously on weekly basis for four weeks after meningococcal vaccination. His serum creatinine stabilized after eculizumab administration between 2.60-3.00 mg/dL. Due to a lack of viable options, as well as a poor performance status, the patient opted for palliative care and died four months later.

## Discussion

There are two factors that give rise to TMA in patients with an underlying malignancy (Table 1). First, the malignancy itself can cause TMA, either via metastases of the microvasculature or direct spread to the bone marrow. Also, different chemotherapy regimens, including gemcitabine, cisplatin, bevacizumab, and mitomycin-C can induce TMA either through direct endothelial damage or by an immune-mediated mechanism with the development of drug-dependent autoantibodies [4-5].

**Table 1:** Mechanism of TMA in malignancy Source: [5] MAHA: microangiopathic hemolytic anemia; TMA: thrombotic microangiopathy

Cause of TMA	MAHA and Thrombocytopenia	Mechanism
Cancer-induced TMA	Present	Systemic microvascular metastases and bone marrow metastases or necrosis
Chemotherapy-induced TMA	Present	Dose-dependent toxicity and drug-dependent antibody reaction

Discerning malignancy-induced TMA versus medication-induced TMA is important for managing the patient appropriately [5]. Symptoms such as weakness, weight loss, cough, shortness of breath, and pain are commonly associated in patients with cancer-induced TMA [5]. Unusual findings of MAHA and thrombocytopenia without evidence of any end-organ damage are also important clues in considering the development of TMA. Our patient with pancreatic cancer suffered from gemcitabine-induced hemolytic uremic syndrome (GiHUS).

GiHUS should be suspected in a patient with malignancy when renal dysfunction occurs with no obvious cause. aHUS may not present at acute onset with all classic signs (i.e. thrombocytopenia, MAHA, acute renal failure). The diagnosis of aHUS can be challenging given the significant clinical overlap of the various TMAs (Table 2).

**Table 2:** Laboratory features of the three forms of thrombotic microangiopathy are presented Source: [6] aHUS: atypical hemolytic uremic syndrome; TTP: thrombotic thrombocytopenic purpura; STEC-HUS: Shiga toxin-producing Escherichia coli hemolytic uremic syndrome

Anemia	Thrombocytopenia	Target organ injury	ADAMTS 13	Shiga-Toxin	Diagnosis
Present	Present	Present	>5%	Negative	aHUS
Present	Present	Present	<5%	Negative	TTP
Present	Present	Present	Normal	Positive	STEC -HUS

The incidence of GiHUS has been reported to be between 0.02% and 2.2% [7-8]. The removal of the causative agent from the treatment is often sufficient to resolve abnormal hematological and renal parameters. Our patient did not demonstrate any substantial improvement despite being off the offending chemotherapeutic agent for one month.

Eculizumab, a complement component inhibitor, which binds to C5, blocks the generation of proinflammatory C5a and C5b-9 in the complement system. It has been shown to be effective in treating chemotherapy-induced TMA or aHUS [9]. A report of one case series on four patients with GiHUS who progressed despite stopping chemotherapy showed that eculizumab proved effective in all four patients. Their anemia and renal function improved, with no adverse events [9]. In our patient, treatment with eculizumab resulted in some improvement in his kidney function, likely due to the dysregulation of the complement pathway. Eculizumab has been approved for the treatment of paroxysmal nocturnal hemoglobinuria (PNH), aHUS, and refractory generalized myasthenia gravis (gMG) [10]. This medication is associated with certain risks, one of them being the increased susceptibility to infection with Neisseria meningitidis or meningococcus [11]. All patients must be vaccinated with a meningococcal vaccine before beginning treatment. The potential long-term effects of this medication, including hepatotoxicity, have been reported [12-13]. So far, many trials have been conducted to study the effect of improved and alternative complement inhibitors. Phase III trials are currently ongoing to study the long-acting variant of eculizumab-ravulizumab in patients with PNH and aHUS [14-15].

## Conclusions

In patients receiving gemcitabine for any malignancies, either therapeutic or palliative, clinicians should have a low threshold for suspecting HUS in the presence of worsening anemia, thrombocytopenia, and/or renal function. Eculizumab has been approved since 2011 in many developed countries for the treatment of aHUS, but its use is limited due to cost, unknown treatment duration, and vague dose intervals to keep patients in remission. New complement-directed therapeutics and the development of biomarkers that predict preclinical TMA should be the main drivers in preventing severe complement-related disorders.
